# Discovery and Characterization of a Potent and Selective Inhibitor of *Aedes aegypti* Inward Rectifier Potassium Channels

**DOI:** 10.1371/journal.pone.0110772

**Published:** 2014-11-06

**Authors:** Rene Raphemot, Matthew F. Rouhier, Daniel R. Swale, Emily Days, C. David Weaver, Kimberly M. Lovell, Leah C. Konkel, Darren W. Engers, Sean F. Bollinger, Corey Hopkins, Peter M. Piermarini, Jerod S. Denton

**Affiliations:** 1 Department of Anesthesiology, Vanderbilt University Medical Center, Nashville, TN, United States of America; 2 Department of Pharmacology, Vanderbilt University School of Medicine, Nashville, TN, United States of America; 3 Department of Entomology, Ohio Agricultural Research and Development Center, The Ohio State University, Wooster, OH, United States of America; 4 Institute of Chemical Biology, Vanderbilt University School of Medicine, Nashville, TN, United States of America; 5 Institute for Global Health, Vanderbilt University, Nashville, TN, United States of America; 6 Department of Chemistry, Vanderbilt University School of Medicine, Nashville TN, United States of America; United States Department of Agriculture, Beltsville Agricultural Research Center, United States of America

## Abstract

Vector-borne diseases such as dengue fever and malaria, which are transmitted by infected female mosquitoes, affect nearly half of the world's population. The emergence of insecticide-resistant mosquito populations is reducing the effectiveness of conventional insecticides and threatening current vector control strategies, which has created an urgent need to identify new molecular targets against which novel classes of insecticides can be developed. We previously demonstrated that small molecule inhibitors of mammalian Kir channels represent promising chemicals for new mosquitocide development. In this study, high-throughput screening of approximately 30,000 chemically diverse small-molecules was employed to discover potent and selective inhibitors of *Aedes aegypti* Kir1 (*Ae*Kir1) channels heterologously expressed in HEK293 cells. Of 283 confirmed screening ‘hits’, the small-molecule inhibitor VU625 was selected for lead optimization and in vivo studies based on its potency and selectivity toward *Ae*Kir1, and tractability for medicinal chemistry. In patch clamp electrophysiology experiments of HEK293 cells, VU625 inhibits *Ae*Kir1 with an IC_50_ value of 96.8 nM, making VU625 the most potent inhibitor of *Ae*Kir1 described to date. Furthermore, electrophysiology experiments in *Xenopus* oocytes revealed that VU625 is a weak inhibitor of *Ae*Kir2B. Surprisingly, injection of VU625 failed to elicit significant effects on mosquito behavior, urine excretion, or survival. However, when co-injected with probenecid, VU625 inhibited the excretory capacity of mosquitoes and was toxic, suggesting that the compound is a substrate of organic anion and/or ATP-binding cassette (ABC) transporters. The dose-toxicity relationship of VU625 (when co-injected with probenecid) is biphasic, which is consistent with the molecule inhibiting both *Ae*Kir1 and *Ae*Kir2B with different potencies. This study demonstrates proof-of-concept that potent and highly selective inhibitors of mosquito Kir channels can be developed using conventional drug discovery approaches. Furthermore, it reinforces the notion that the physical and chemical properties that determine a compound's bioavailability in vivo will be critical in determining the efficacy of Kir channel inhibitors as insecticides.

## Introduction

Mosquitoes are vectors of protozoan, filarial nematode, and viral pathogens that cause numerous human diseases, including malaria, lymphatic filariasis, and dengue fever. These diseases impose an enormous burden on global health and profoundly impair socioeconomic advancement in developing countries [1]. The overuse of a limited number of insecticides has led to the emergence of insecticide-resistant populations of mosquitoes, which is hampering the effectiveness of vector control efforts [2], [3], [4]. Consequently, there is a need to identify new molecular targets against which insecticides can be developed and deployed.

An emerging body of evidence from our group supports the idea that inward rectifier potassium (Kir) channels represent viable targets for insecticide development [5], [6], [7]. Kir channels are tetrameric proteins that conduct K^+^ ions across the cell membrane and thereby generate an ionic current that underlies various cellular functions. Dipteran insects possess three major Kir channel subtypes, denoted Kir1, Kir2 and Kir3. In *Drosophila melanogaster*, there are three genes that encode Kir channels (*Dr*Kir1, *Dr*Kir2, *Dr*Kir3), which play important roles in osmoregulation, immunity, and development [8], [9], [10], [11]. In *Aedes aegypti*, there are five Kir channel genes (*AeKir1, AeKir2A, AeKir2B, AeKir2B*’ and *AeKir3*), which are expressed in various body segments and tissues such as the carcass (thorax and abdomen), head, Malpighian tubules, midgut, and hindgut [6], [12]. We showed previously in vitro that the *A. aegypti* Kir1 (*Ae*Kir1) channel mediates strong inward rectifying K^+^ currents that are blocked by barium and the small molecule inhibitors, VU573 and VU590 [7], [12], [13]. Moreover, a hemolymph injection of either VU573 or VU590 inhibits the excretion of urine by adult female mosquitoes, leads to abdominal bloating, and incapacitates mosquitoes within 24 h [5].

Taken together, the above studies indicate that Kir channels represent promising molecular targets for insecticides that have a novel mechanism of action by disrupting the renal-dependent regulation of extracellular fluid homeostasis (i.e., renal failure). However, in mammals, Kir channels regulate the electrical excitability of neurons and cardiac cells, hormone secretion, and transport of K^+^ ions across epithelial tissues of the kidney and gut [14]. Missense mutations that perturb the activity of Kir channels cause human diseases of the heart, nervous system, pancreas, and kidney [15], [16], [17]. Thus, efforts aimed at developing insecticides to target Kir channels must verify that lead compounds do not perturb the functions of mammalian Kir channels.

As such, the above ‘tool’ compounds VU573 and VU590 allowed us to establish proof-of-concept, but are not suitable for insecticide development, in part, because they inhibit mammalian Kir channels with greater potency than *Ae*Kir1 [18], [19]. Here, we aim to discover new chemical probes of *Ae*Kir1 channels that exhibit improved potency and selectivity compared to the tool compounds by optimizing and validating an existing fluorescent thallium (Tl^+^) flux-based assay of *Ae*Kir1 function [5] for high-throughput screening (HTS) of small molecule libraries. Screening approximately 30,000 small molecules from the chemical library of the Vanderbilt Institute of Chemical Biology (VICB) resulted in the identification of 283 compounds with activity against *Ae*Kir1 channels. We focus on the in vitro and in vivo activity of one of these compounds, N-(3-methoxyphenyl)-2-methyl-1-propionylindoline-5-sulfonamide (VU625), which exhibits nanomolar affinity and is highly selective for *Ae*Kir1 over mammalian Kir channels.

## Materials and Methods

### Tl^+^ flux assays

Tl^+^ flux assays were performed essentially as described previously [13], [18], [19]. Briefly, stably transfected T-Rex-HEK-293 cells expressing *Ae*Kir1 channels were cultured overnight in 384-well plates (20,000 cells/20 µL/well black-walled, clear-bottomed BD PureCoat amine-coated plates (BD, Bedford, MA) with a plating media containing DMEM, 10% dialyzed FBS and 1 µg/mL tetracycline. The next day, the cell culture medium was replaced with a dye-loading solution containing assay buffer (Hanks Balanced Salt Solution with 20 mM HEPES, pH 7.3), 0.01% (*w/v*) Pluronic F-127 (Life Technologies, Carlsbad, CA), and 1.2 µM of the thallium-sensitive dye Thallos-AM (TEFlabs, Austin, TX). Following 1 hr incubation at room temperature, the dye-loading solution was washed from the plates and replaced with 20 µL/well of assay buffer.

The plates were transferred to a Hamamatsu Functional Drug Screening System 6000 (FDSS6000; Hamamatsu, Hamamatsu (or Bridgewater, NJ), Japan) where 20 µL/well of test compounds in assay buffer (as prepared below) were added and allowed to incubate with the cells for 20 min. After the incubation period, a baseline recording was collected at 1 Hz for 10 s (excitation 470±20 nm, emission 540±30 nm) followed by a Tl^+^ stimulus buffer addition (10 µL/well) and data collection for an additional 4 min. The Tl^+^ stimulus buffer contains in (mM) 125 NaHCO_3_, 1.8 CaSO_4_, 1 MgSO_4_, 5 glucose, 12 Tl_2_SO_4_, 10 HEPES, pH 7.4. For Tl^+^ flux assays on the mammalian channels Kir2.x, Kir4.1 and Kir6.2/SUR1 expressing cells, the Tl^+^ stimulus buffer contained 1.8 mM Tl_2_SO_4_. Also, Tl^+^ flux assays on Kir3.1/3.2/mGlu8 expressing cell, required addition of an EC_80_ concentration of glutamate (Sigma-Aldrich, St. Louis, MO) with the Tl^+^ stimulus buffer [19].

The test compounds were transferred to daughter polypropylene 384-well plates (Greiner Bio-One, Monroe, NC) using an Echo555 liquid handler (Labcyte, Sunnyvale, CA), and then diluted into assay buffer to generate a 2X stock in 0.6% DMSO (0.3% final). For Tl^+^ flux assays on Kir6.2/SUR1 expressing cells, test compounds were diluted in assay buffer containing diazoxide (250 µM final) to induce channel activation [20]. Concentration-response curves (CRCs) were generated by screening compounds at 3-fold dilution series in 4-point (1 µM–30 µM) or 11-point (1 nM–30 µM) CRCs.

Tl^+^ flux data were analyzed as previously described [19], [21], [22] using a combination of Excel (Microsoft Corp, Redmond, WA) with XLfit add-in (IDBS, Guildford, Surrey, UK) and OriginPro (OriginLab, Northampton, MA) software. Raw data were opened in Excel and each data point in a given trace was divided by the first data point from that trace (static ratio) followed by subtraction of data points from control traces generated in the presence of vehicle controls. The slope of the fluorescence increase beginning 5 s after Tl^+^ addition and ending 15 s after Tl^+^ addition was calculated.

### Compound synthesis

#### 2,2,2-trifluoro-1-(2-methylindolin-1-yl)ethan-1-one

The reagents and conditions are illustrated in Figure S1. To a round bottom flask equipped with a magnetic stir bar, 2-methylindoline (4.8 mL, 37 mmol, 1 eq.) and pyridine (46 mL) were added. The reaction mixture was cooled to 0°C and trifluoroacetic anhydride (6.3 mL, 44 mmol, 1.2 eq.) was added dropwise. The reaction mixture was allowed to warm to room temperature and was stirred an additional 2 hours. The reaction was quenched with water (50 mL) and diluted with DCM (100 mL). The organic layer was separated and washed subsequently with water (50 ml) and brine (50 mL), dried over Na_2_SO_4_, and concentrated under reduced pressure. The crude material (8.33g, 98%) was used without purification. LCMS: R_T_ = 0.785 min, [M+H]^+^ = 229.6; >98%.

**2-methyl-1-(2,2,2-trifluoroacetyl)indoline-5-sulfonyl chloride**: Chlorosulfonic acid (22 mL, 330 mmol, 9 equiv.) was added to a 100 mL round bottom flask equipped with a reflux condensor, and cooled to 0°C. To this, 2,2,2-trifluoro-1-(2-methylindolin-1-yl)ethan-1-one, (8.5 g, 37 mmol, 1 eq.) was added dropwise. The reaction mixture was removed from the ice bath. The vial was heated to 40°C for 1 hour. The reaction was subsequently cooled to room temperature and PCl_5_ (7.7 g, 37 mmol, 1 equiv.) was added slowly. After gas evolution ceased, the reaction mixture was heated to 80°C for 1 hour. The reaction mixture was cooled to room temperature and then placed in an ice bath. Water was added very slowly to the reaction mixture. Subsequently, DCM was added and the reaction was filtered through a phase separator. The organic layer was concentrated under reduced pressure and used without subsequent purification (6.46 g, 53%).

**N-(3-**methoxyphenyl**)-2-methylindoline-5-sulfonamide**: 2-methyl-1-(2,2,2-trifluoroacetyl)indoline-5-sulfonyl chloride (2.5 g, 7.6 mmol, 1 eq.) was diluted with DCM (10 mL). 3-methoxyaniline (1.71 mL, 15.2 mmol, 2 eq.) followed by *N*,*N*-Diisopropylethylamine (5.3 mL, 31 mmol, 4 eq.) was added to the reaction. Reaction progress was monitored by LCMS. Once the reaction was deemed complete, it was diluted with DCM (40 mL) and washed with water (2x, 50 mL) and brine (50 mL). The organic layer was dried over Na_2_SO_4_ and concentrated under reduced pressure. Purification by flash chromatography (0%–100% EtOAc in Hexanes) afforded the desired product (2.66 g, 85%). LCMS: R_T_ = 0.800 min., [M+H]^+^ = 414.7; >98% @ 220 and @ 254 nm. The trifluoroacetate was removed by stirring in a 1∶1∶1 mixture of MeOH, THF, and 10% NaOH affording the title compound (782 mg, 38%). LCMS: R_T_ = 0.665 min., [M+H]^+^ = 318.8; >98% @ 220 and @ 254 nm.

**N-(3-**methoxyphenyl**)-2-methyl-1-propionylindoline-5-sulfonamide (VU0077625)**: N-(3-methoxyphenyl)-2-methylindoline-5-sulfonamide (11 mg, 0.035 mmol, 1 eq.) was diluted with DCM (0.3 mL). To this reaction, pyridine was added (0.011 mL, 0.14 mmol, 4 eq.) followed by propionyl chloride (0.003 mL, 0.05 mmol, 1.5 eq.). Reaction progress was monitored by LCMS. Once the reaction was deemed complete it was concentrated under forced air and heat and was subsequently purified on preparative HPLC (3 mg, 26%). ^1^H NMR (400.1 MHz, CDCl_3_) δ ppm): 8.17 (bs, 1 H); 7.67 (dd, *J* = 1.69, 8.72 Hz, 1 H); 7.58 (s, 1 H); 7.16 (t, *J* = 8.25 Hz, 1 H); 6.69–6.57 (m, 4 H); 4.58 (bs, 1 H); 3.74 (s, 3 H); 3.38–3.32 (m, 1 H); 2.66–2.47 (m, 4 H); 1.29–1.22 (m, 5 H). HRMS (TOF, ES^+^) C_19_H_23_N_2_O_4_S [M+H]^+^ calc'd for 375.1379, found 375.1381.

### Patch clamp electrophysiology

T-REx-HEK293-*Ae*Kir1 cells were voltage clamped in the whole-cell configuration of the patch clamp technique after overnight induction with tetracycline (1 µg/ml) essentially as described earlier [5]. Briefly, patch electrodes were pulled from silanized 1.5 mm outer diameter borosilicate microhematocrit tubes using a Narishige PC-10 two-stage puller. Electrode resistance ranged from 3.5 to 5.5 MΩ when filled with the following intracellular solution (in mM): 135 KCl, 2 MgCl_2_, 1 EGTA, 10 HEPES free acid, 2 Na_2_ATP (Roche, Indianapolis, IN), pH 7.3, 275 mOsm. The standard bath solution contained (in mM): 135 NaCl, 5 KCl, 2 CaCl_2_, 1 MgCl_2_, 5 glucose, 10 HEPES free acid, pH 7.4, 290 mOsm. Whole-cell currents were recorded under voltage-clamp conditions using an Axopatch 200B amplifier (Molecular Devices, Sunnyvale, CA). Electrical connections to the amplifier were made using Ag/AgCl wires and 3 M KCl/agar bridges. Electrophysiological data were collected at 5 kHz and filtered at 1 kHz. Data acquisition and analysis were performed using pClamp 9.2 software (Axon Instruments). All recordings were made at room temperature (20–23°C).

### Heterologous expression of *Ae*Kir1 and *Ae*Kir2B in Xenopus oocytes

*Ae*Kir1 and *Ae*Kir2B channels were expressed heterologously in *Xenopus laevis* oocytes as described previously [6]. In brief, defolliculated *Xenopus* oocytes (purchased from Ecocyte Bioscience, Austin, TX) were injected with 10 ng (0.35 ng/nL) of either *Ae*Kir1 or *Ae*Kir2B cRNA and cultured for 3–7 days in OR3 media at 18°C. Oocytes injected with 28 nl of nuclease-free H_2_O served as controls.

### Electrophysiology of Xenopus oocytes

All electrophysiological experiments on *Xenopus* oocytes were performed at room temperature. The compositions of the solutions used in these experiments are shown in Table 1. When present, VU625 was dissolved in solution *III* or solution *V* to a final concentration of 0.1, 1, 5, 15, or 50 µM (0.05% DMSO). All solutions were delivered by gravity to a RC-3Z oocyte chamber (Warner Instruments, Hamden, CT) via polyethylene tubing at a flow rate of ∼2 ml/min. Solution changes were made with a Rheodyne Teflon 8-way Rotary valve (Model 5012, Rheodyne, Rohnert Park, CA).

**Table 1:** Compositions (in mM) of solutions used in *Xenopus* oocyte electrophysiology.

Solution #	*I*	*II*	*III*	*IV*	*V*
NaCl	96	88.5	88.5	73.5	73.5
NMDG-Cl	0	9.5	0	24.5	0
KCl	2	0.5	10	0.5	25
MgCl_2_	1.0	1.0	1.0	1.0	1.0
CaCl_2_	1.8	1.8	1.8	1.8	1.8
HEPES	5	5	5	5	5

The pH of all solutions was adjusted to 7.5 with NMDG-OH.

The osmolality of each solution was verified to be 190 mOsm kg^−1^ H_2_O (±5 mOsm kg^−1^ H_2_O) by vapor pressure osmometry.

NMDG = N-methyl-D-glucamine.

Electrophysiological recordings from oocytes were conducted as described previously [6] In brief, each oocyte was transferred to the holding chamber under superfusion with solution *I* and impaled with two conventional-glass microelectrodes backfilled with 3 M KCl (resistances of 0.5–1.5 MΩ) to measure membrane potential (V_m_) and whole-cell membrane current (I_m_), respectively. Current-voltage (I–V) relationships of oocytes were acquired as described previously [6]. In brief, the oocytes were subjected to a voltage-stepping protocol consisting of 20 mV steps from −140 mV to +40 mV (100 ms each). After the conclusion of the voltage-stepping protocol, the clamp was turned off and a new solution was superfused through the chamber for ∼90 s before acquiring another I–V relationship. All V_m_ and I_m_ values were recorded by a Digidata 1440A Data Acquisition System (Molecular Devices) and the Clampex module of pCLAMP. The I–V plots were generated using the Clampfit module of pCLAMP.

To evaluate the inhibition of *Ae*Kir1 and *Ae*Kir2B activity by VU625, we focused on the maximal inward currents elicited by the voltage-stepping protocol, which occur at a voltage of −140 mV. For *Ae*Kir1 oocytes, the background, inward currents in solution *II* (i.e., 0.5 mM K^+^) were subtracted from those in 1) solution *III* (i.e., 10 mM K^+^) to calculate the total inward current for an oocyte before exposure to VU625 (I_A_), and 2) solution *III* with VU625 to calculate the inward current after exposure to the small molecule (I_B_). The percent inhibition of the inward current was calculated by subtracting I_B_ from I_A_ and then dividing by I_A_. For *Ae*Kir2B oocytes, a similar protocol was followed and similar calculations were made, except solution *IV* replaced solution *II* and solution *V* replaced solution *III*.

### Mosquito colony

The *Aedes aegypti* mosquito colony used in the present study is identical to that described previously [6]. As before, only adult female mosquitoes 3–10 days post emergence were utilized for experiments.

### Mosquito toxicology experiments

Adult female mosquitoes for injection were anesthetized on ice and impaled through the metapleuron using a pulled-glass capillary attached to a nanoliter injector (Nanoject II, Drummond Scientific Company, Broomall, PA). Each mosquito received a single hemolymph injection of 69 nL of solution. The injection solution consisted of a potassium-rich phosphate buffered saline (K^+^-PBS), 15% DMSO, 1% β-cyclodextrin, 0.1% Solutol, and a concentration of VU625 to deliver the doses indicated. In experiments where probenecid was used, water-soluble probenecid (Biotium, Hayward CA) was included in the injection solution at 50 mM, thereby providing a dose of 3.4 nmol per mosquito.

The K^+^-PBS solution consisted of the following in mM: 92.2 NaCl, 47.5 KCl, 10 Na_2_HPO_4_, and 2 KH_2_PO_4_ (pH 7.5). A total of 10 mosquitoes were injected for a given treatment or dose, and then were placed into small cages within a rearing chamber (28°C, 80% relative humidity, 12∶12 light:dark) and allowed free access to a solution of 10% sucrose. The mosquitoes were observed at 24 hr after injection. For each treatment, 3–7 replicates of 10 mosquitoes each were performed.

### Mosquito excretion experiments

The excretory capacity of mosquitoes was measured as described [6]. In brief, after anesthetizing mosquitoes on ice, their hemolymph was injected as described above with 900 nL of a K^+^-PBS vehicle containing 1.15% DMSO, 0.077% β-cyclodextrin, and 0.008% Solutol, or the vehicle containing VU625 (0.77 mM) to deliver a dose of 690 pmol of VU625 per mosquito. In experiments where probenecid was used, the vehicle was supplemented with water- soluble probenecid (3.08 mM) to deliver a dose of 3.4 nmol of probenecid per mosquito. After injection, the mosquitoes were placed immediately in a graduated, packed-cell volume tube (MidSci, St. Louis, MO; 5 mosquitoes per tube) and held at 28°C. The volume of urine excreted at 60 min post injection was measured as described previously [6], and all mosquitoes were confirmed to be alive at the end of 60 min period. For each treatment, 6–18 independent trials of 5 mosquitoes per treatment were performed.

### Statistical analyses

#### Tl+ flux assay

The Z′ value was calculated as described earlier [21], using the following formula: 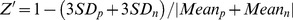
where SD is standard deviation, p and n are vehicle control and compound inhibited flux values respectively.

To compare the effect of DMSO on *Ae*Kir1-mediated Tl^+^ flux, a one-way ANOVA was performed with a Tukey's multiple comparison test. Prism software (GraphPad Software) was used to generate CRC from Tl^+^ flux. Half-inhibition concentration (IC_50_) values were calculated from fits using a four parameter logistic equation.

#### Mosquito toxicology and urine excretion

Prism (GraphPad Software) was used to generate a dose-response curve for the toxicity of VU625; the doses (x-axis) were first log transformed and then the mortality data was normalized using Prism, where the smallest value and largest values in a data set equal ‘0%’ and ‘100%’, respectively. The data were then fitted using a ‘biphasic’ algorithm (<100 constraint) to calculate potencies (ED_25_ and ED_75_ values). To compare 1) the toxic effects among the vehicle, probenecid, VU625, and VU625 + probenecid treatments, and 2) the excretory capacity among the vehicle, probenecid, VU625, and VU625 + probenecid treatments, one-way ANOVAs were performed with Newman-Keuls posttests.

## Results

### Discovery of novel *Ae*Kir1 inhibitors via HTS

**Figure 1:**
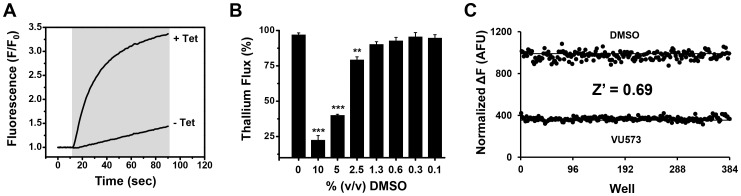
Tl^+^ flux assay of *Ae*Kir1 channel activity for high-throughput screening. (A) Representative Tl^+^-induced changes in Thallos fluorescence in T-Rex-HEK293-*Ae*Kir1 cells cultured overnight with (+Tet) or without (-Tet) tetracycline. The shaded box indicates the cell exposure to Tl^+^. (B) DMSO concentrations up to 1.3% v/v DMSO have no effect on Tl^+^ flux through *Ae*Kir1. Data are means ±SEM (*n* = 3). One-way ANOVA P<0.0001, and asterisks (**, ***) indicate P<0.01 or P<0.001 respectively, when compared to 0% DMSO (Tukey's test). (C) Representative checkerboard analysis using 100 µM VU573 or 0.1% v/v DMSO as the vehicle control. The mean peak fluorescence amplitude of each sample population is indicated with a solid line and alternating samples for DMSO (top) and VU573 (bottom) are graphed as individual points. The mean ±SD Z′ calculated over 6 plates on 3 separate days was 0.69±0.05.

In an effort to discover mosquito-specific inhibitors of *Ae*Kir1, we optimized a Tl^+^ flux assay for HTS of large libraries of chemically diverse small molecules. The assay utilizes a monoclonal T-REx-HEK293 cell line that expresses *Ae*Kir1 from a tetracycline-inducible promoter [5]. The fluorescent dye, Thallos, is used to report the inward flux of Tl^+^ through the *Ae*Kir1 channel pore in a population of cells plated in individual wells of a 384-well plate. As shown in Figure 1A, overnight induction of *Ae*Kir1 expression with tetracycline leads to a robust Tl^+^ flux compared to control cells that were not treated with tetracycline. This assay enables more than 300 compounds to be tested simultaneously in a single plate, and thousands of compounds to be tested daily, for effects on *Ae*Kir1 activity.

The assay was validated for HTS by meeting a series of performance benchmarks. First, the assay was tested for its tolerance to the small-molecule vehicle DMSO at concentrations up to 10% v/v. As shown in Fig. 1B, the Tl^+^-flux mediated by *Ae*Kir1 is unaffected by DMSO concentrations up to 1.3% v/v as compared to the 0% DMSO control (one-way ANOVA, P <0.0001). Next, the assay was tested for uniformity and reproducibility of HTS performance. As shown in Fig. 1C, the average Z′ statistic for these experiments was 0.69±0.05 (Z′≥0.5 is suitable for HTS), indicating that the assay is robust and will enable modulators of *Ae*Kir1 to be identified in HTS with a low false-positive rate.

Approximately 30,000 compounds from the VICB library were screened at a nominal concentration of 10 µM for inhibition of *Ae*Kir1. From this primary screen and following confirmation testing in tetracycline-induced and uninduced T-REx-HEK293-*Ae*Kir1 cells (see Methods), 283 authentic channel-dependent modulators were selected for further study. Because our ultimate goal is to develop Kir channel inhibitors that are active against mosquitoes and not humans, these ‘hits’ were subsequently tested for dose-dependent activity against a panel of mammalian Kir channels, which included Kir1.1, Kir2.1, Kir2.2, Kir2.3, Kir3.1/3.2, Kir4.1, Kir7.1(M125R), and Kir6.2/SUR1 [18], [19], [21]. Four-point concentration response curves (CRCs) were generated for the 283 compounds, resulting in 17 inhibitors with 11 unique chemical scaffolds that exhibited dose-dependent inhibition of *Ae*Kir1 with IC_50_ values below 5 µM and little to no activity (IC_50_≥30 µM) against mammalian Kir channels (data not shown). These compounds were subsequently purchased from commercial vendors, freshly dissolved in DMSO, and assayed in 11-point CRCs against *Ae*Kir1 via the Tl^+^-flux assay.

### VU625 is a potent and preferential inhibitor of *Ae*Kir1 vs. mammalian Kir and *Ae*Kir2B channels

**Figure 2:**
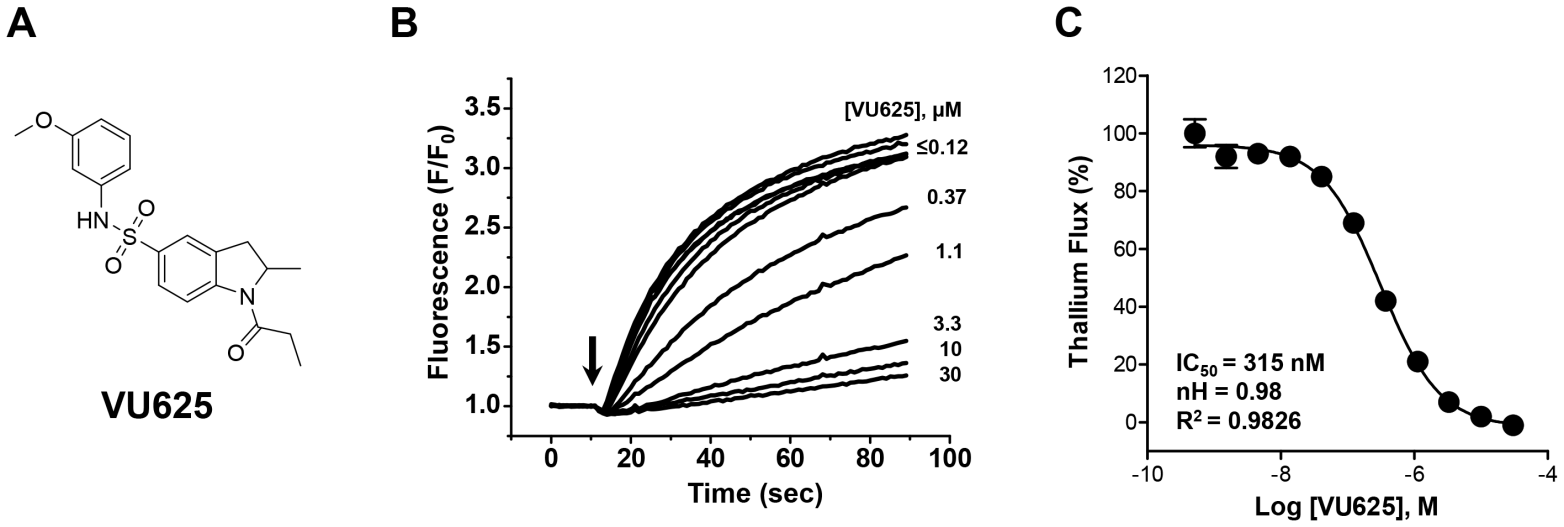
VU625 is a potent inhibitor of *Ae*Kir1 in Tl^+^ flux assays. (A) Chemical structure of VU625. (B) Dose-dependent inhibition of the *Ae*Kir1-mediated Tl^+^ flux by VU625 with concentrations ranging from ≤0.12 to 30 µM. The arrow indicates when Tl^+^ was added to the extracellular bath. (C) Concentration-response curves of VU625 derived from Tl^+^ flux assays. The IC_50_ and Hill-coefficient (nH) values are 315 nM (95% CI: 254.4–390.2 nM) and 0.98 respectively. Data are mean ±SEM. *n* = 4 independent experiments performed in triplicate.

From the aforementioned Tl^+^ flux assays, one compound—N-(3-methoxyphenyl)-2-methyl-1-propionylindoline-5-sulfonamide, termed VU625—was found to inhibit *Ae*Kir1 in 11-point CRCs with an IC_50_ of 0.32 µM (95% CI: 0.25–0.39 µM) and a Hill coefficient value of 0.98 (95% CI: 0.8–1.2) (Fig. 2A–C). VU625 also had no significant effects on the mammalian Kir channels assayed via Tl^+^ flux with the exception of G-protein coupled Kir channels comprised of Kir3.1/3.2 subunits (IC_50_ = 8.6 µM; Table S1). Furthermore, in radioligand displacement assays against 68 mammalian GPCR's, ion channels, and transporters, 10 µM VU625 was active (defined as>50% ligand displacement) against only three targets: adenosine A1 receptor (76% displacement), melatonin MT1 receptor (56% displacement) and 5-HT_2B_ receptor (69% displacement) (Table S2).

**Figure 3:**
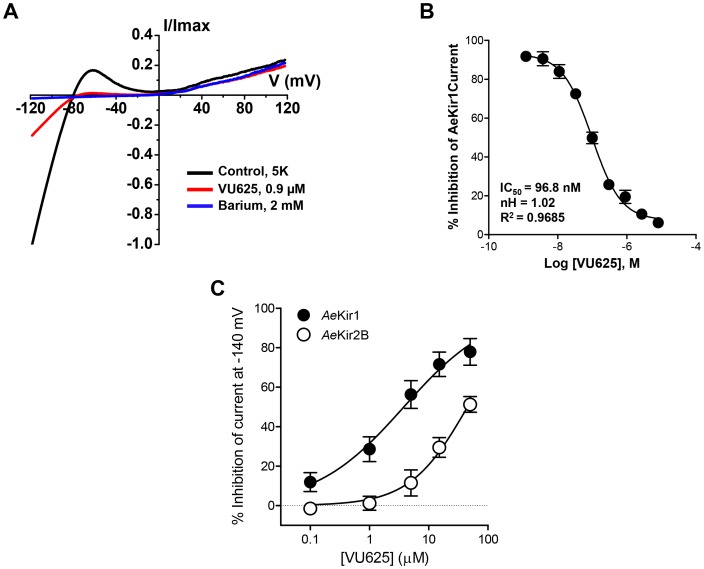
VU625 is a potent and preferential inhibitor of *Ae*Kir1 over *Ae*Kir2B in whole-cell electrophysiology. (A) Normalized *Ae*Kir1 current-voltage relationships obtained from heterologous expression in T-Rex-HEK293 cells, illustrating VU625-dependent inhibition before (control) and after addition of 0.9 µM VU625. Residual *Ae*Kir1 currents were inhibited with 2 mM barium. Cells were voltage clamped at −75 mV and ramped between −120 mV and +60 mV. (B) Concentration-response curve of VU625 derived from patch clamp experiments (*n* = 4–6). The IC_50_ of VU625 is 96.8 nM (95% CI: 75.4–124.2 nM). (C) Concentration-response curves of current inhibition mediated by heterologous expression in *Xenopus* oocytes of *Ae*Kir1 (filled circles) and *Ae*Kir2B (open circles) channels after bath application of VU625. *n* = 4–5 oocytes per concentration. The calculated IC_50_ values of VU625 for *Ae*Kir1 and *Ae*Kir2B current inhibition are 3.8 µM (95% CI: 2.3–6.3 µM) and 45.1 µM (95% CI: 31.7–64.2 µM), respectively.

To further confirm the activity of VU625 obtained from Tl^+^-flux assays, we used patch-clamp electrophysiology to assay the inhibition of *Ae*Kir1 expressed in T-REx-HEK293 cells. In whole-cell patch clamp recordings, VU625 inhibited *Ae*Kir1 channel activity with an IC_50_ of 96.8 nM (95% CI: 75.4–124.2 nM) and a Hill coefficient value of 1.02 (95% CI: 0.8–1.3) (Fig. 3A, B).

In a previous paper, we demonstrated that other small molecule inhibitors of *Ae*Kir1 (i.e., VU573 and VU590) can have different pharmacological effects on *Ae*Kir2B [6]. Thus, we sought to determine the effects of VU625 on *Ae*Kir2B channel activity, utilizing *Xenopus* oocytes heterologously expressing *Ae*Kir2B. *Ae*Kir1 expressing oocytes served as positive controls. Figure 3C shows that VU625 inhibits *Ae*Kir1- and *Ae*Kir2B-mediated K^+^ currents with IC_50_ values of 3.8 µM (95% CI: 2.3–6.3 µM) and 45.1 µM (95% CI: 31.7–64.2 µM), respectively. Thus, VU625 inhibits both *Ae*Kir1 and *Ae*Kir2B channels, albeit with greater affinity for *Ae*Kir1. It should be noted that the reduction in VU625 potency observed in *Xenopus* oocytes compared to HEK cells is typical for a small-molecule inhibitor of Kir channels and has been observed for structurally diverse compounds and Kir channels [5], [6], [19], [23].

### Chemical lead optimization and structure-activity relationships

**Figure 4:**
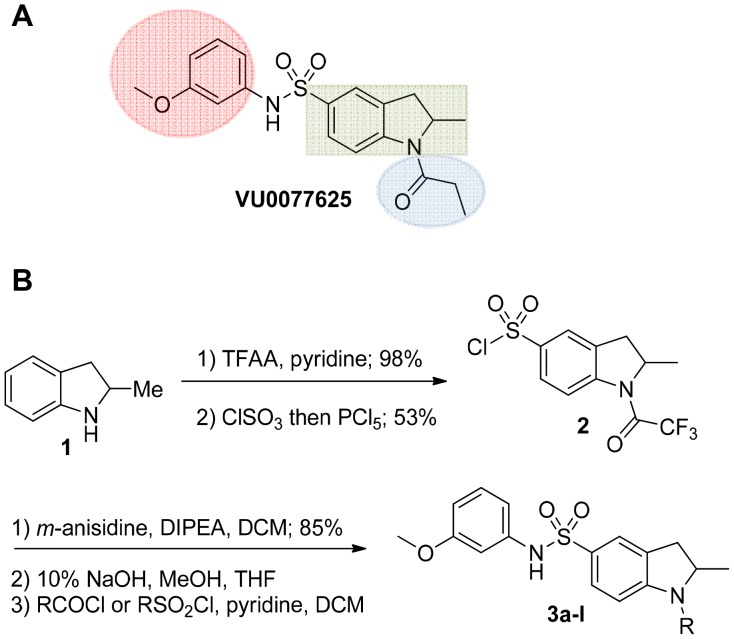
Design and chemical lead optimization strategy for VU625. (A) Modular approach to assess three areas of diversification of VU625: sulfonamide (red shading), central core (green shading), and southern amide (blue shading) portions. (B) General synthetic approach to access VU625 and analogs around the amide and sulfonamide portions.

Because of its potency, clean ancillary pharmacology and chemical tractability (Figures 2–3, Tables S1–S2), VU625 was selected for lead optimization (**3a**, Table 2). We partitioned the compound into three areas for structure-activity relationship (SAR) exploration denoted as the sulfonamide, central core, and southern amide portions (Fig. 4A). The first generation libraries held the sulfonamide and the central core sections constant and diversified the southern amide portion (Table 2). The synthetic scheme (Fig. 4B) for this portion was straightforward and started with protection of the amine with trifluoracetamide (TFAA, pyridine) followed by sulfonyl chloride formation (ClSO_3_, PCl_5_). Next, the sulfonamide was formed, the protecting group was removed, and either the amide or sulfonamide was formed (see Methods for details). Little tolerance for steric bulk was seen in this portion of the molecule. That is, the trifluoroacetamide (VU0477197, **3b**, Table 2) retained potency (0.58 µM), however, larger aromatic amides were much less active (**3c–g**, Table 2). The same trend was observed for the sulfonamide compounds, with smaller sulfonamides retaining nanomolar activity (VU0477691, **3k**, 0.76 µM; VU0477692, **3l**, 0.82 µM) and the larger aromatic group leading to less activity (**3h**, Table 2).

**Table 2:** Structure-activity relationships and lead optimization summary of VU0077625 scaffold.

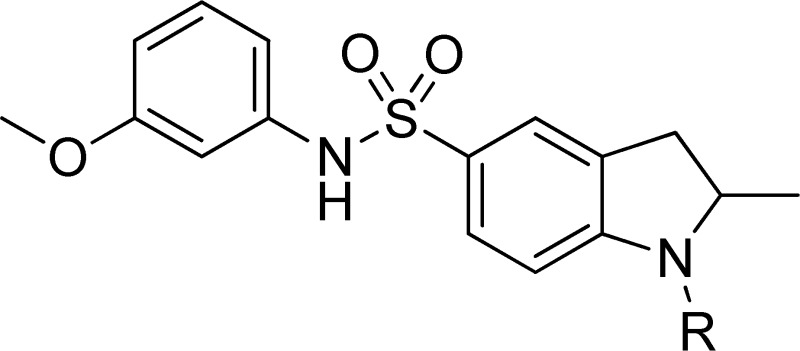			
Cmpd	R	VU#	IC_50_±SEM (µM)
**3a**	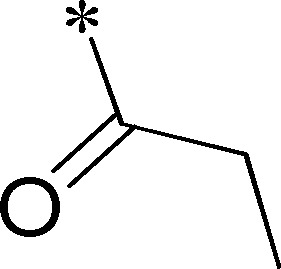	VU0077625	0.36±0.02
**3b**	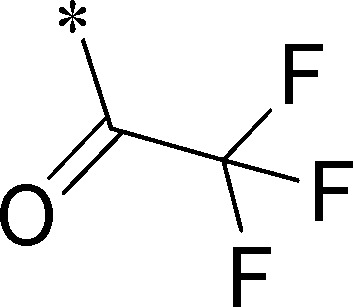	VU0477197	0.58±0.04
**3c**	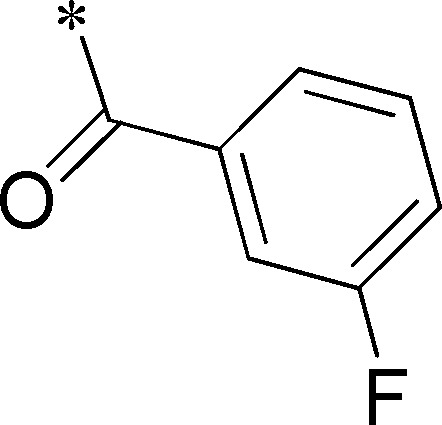	VU0477684	5.20±0.40
**3d**	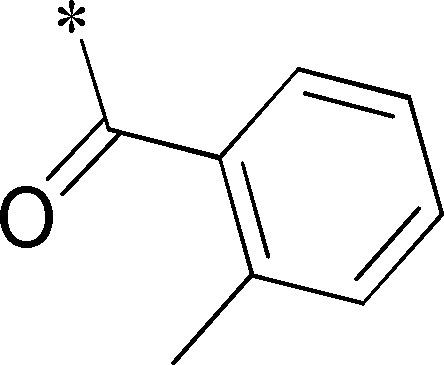	VU0477693	4.41±1.11
**3e**	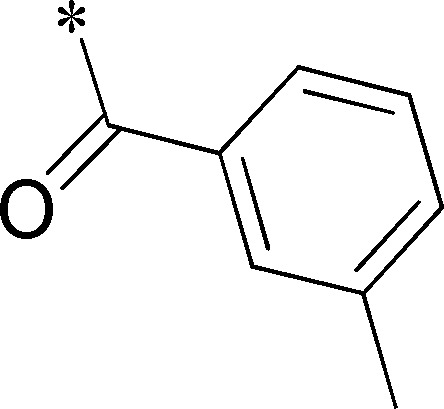	VU0477694	3.87±1.97
**3f**	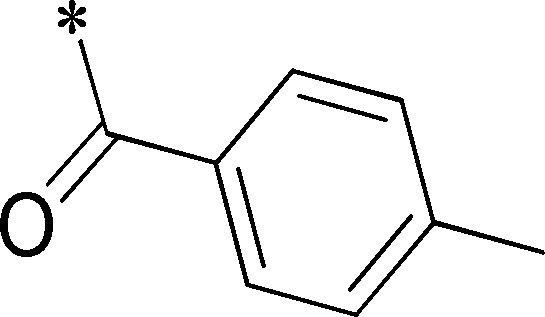	VU0477688	>30
**3g**	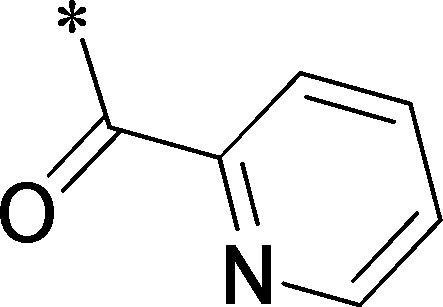	VU0477685	4.40±0.50
**3h**	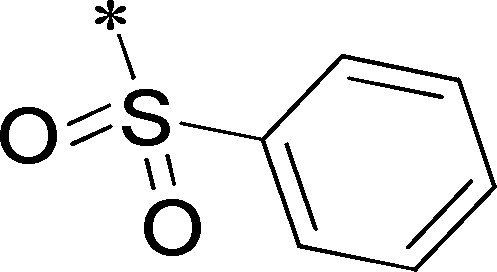	VU0477686	3.29±0.89
**3i**	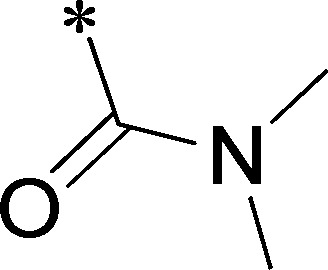	VU0477687	2.09±0.49
**3j**	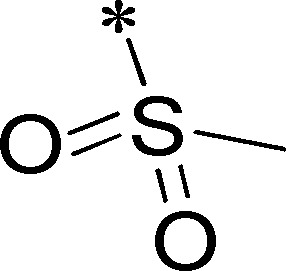	VU0477690	2.82±0.48
**3k**	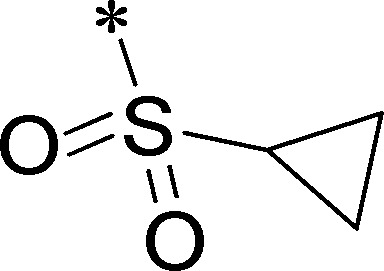	VU0477691	0.76±0.00
**3l**	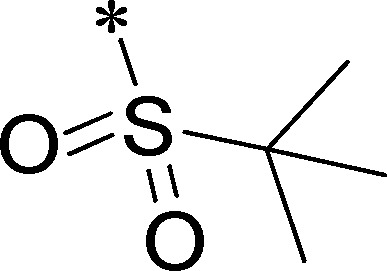	VU0477692	0.82±0.48

IC_50_ values were derived from 11-point CRCs on *Ae*Kir1 in Tl^+^ flux experiments performed in triplicate on two separate days.

Next, we evaluated the left-hand sulfonamide portion of the molecule, however, all efforts to change the 3-methoxyaryl moiety led to significant reductions in potency (see Table S3). Finally, we explored the central core with the intent of establishing the minimal pharmacophore needed for activity against *Ae*Kir1. To this end, the indoline core was replaced with simple aryl, heteroaryl or biaryl groups which all led to compounds with much reduced activity (>10-fold loss of potency). However, an interesting SAR was seen with very closely related 6,6- or 6,5-indole or dihydroquinolinone-like structures (**4a–f**, Table 3). The simple *N*-methyl indole (VU0481807, **4a**, 0.55 µM, Table 3) retained most of the activity as VU625 and addition of a 2-methyl (VU0486620, **4b**, 0.97 µM, Table 3) led to a further minor reduction in activity. Expanding the ring system and addition of a lactam (**4c–e**, Table 3) was not productive. Lastly, removal of the methyl group in the indoline system of VU625, led to a ∼3-fold loss of potency (VU0483404, **4f**, 1.15 µM, Table 3).

**Table 3:** Structure-activity relationships and lead optimization summary for the central core portion of VU0077625 scaffold.

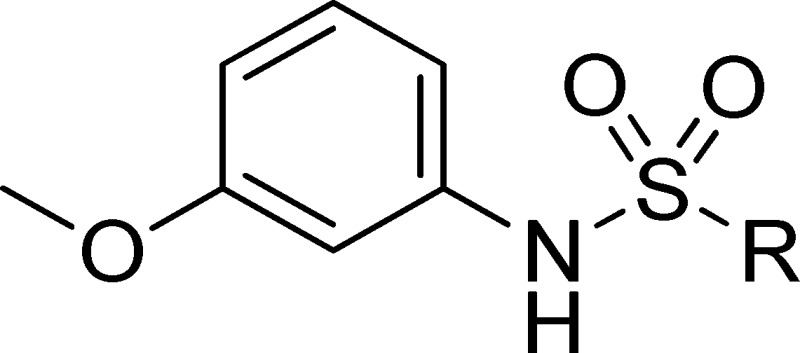			
Cmpd	R	VU#	IC_50_±SEM (µM)
**4a**	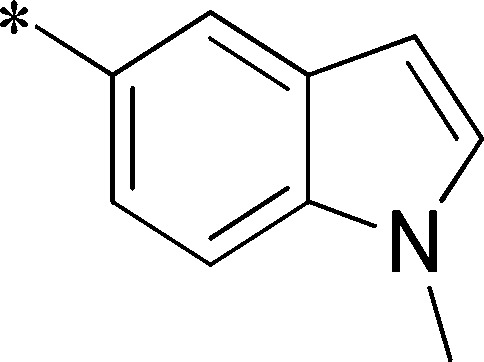	VU0481807	0.55±0.08
**4b**	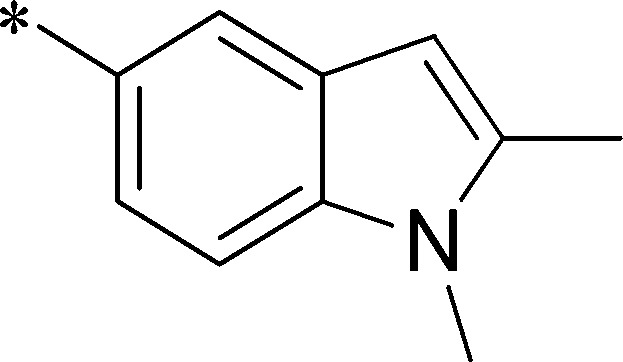	VU0486620	0.97±0.10
**4c**	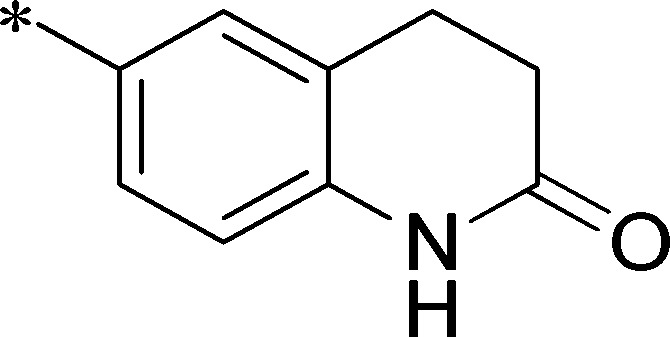	VU0481811	>30
**4d**	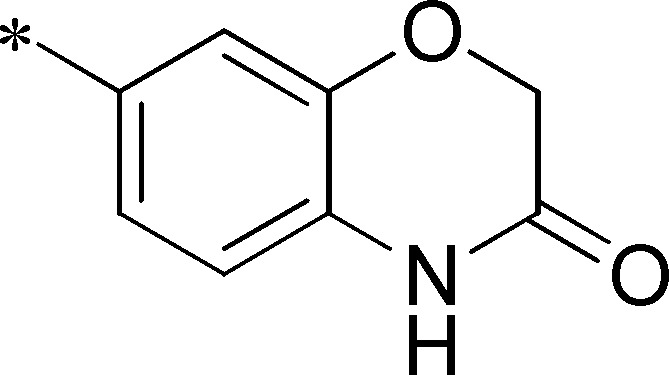	VU0483082	>30
**4e**	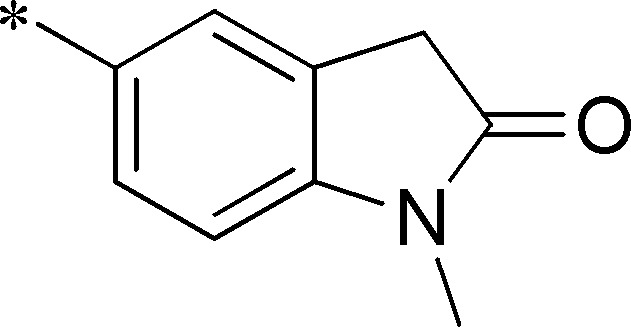	VU0483402	>30
**4f**	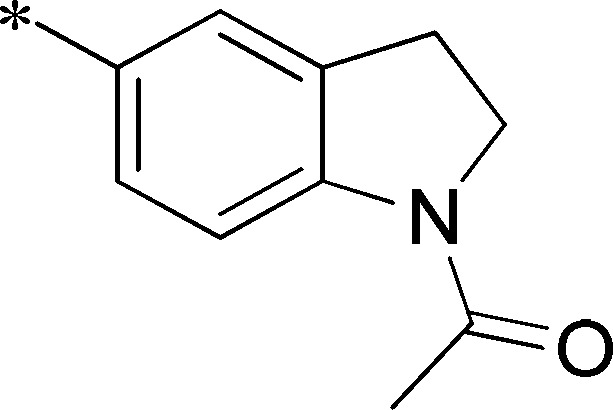	VU0483404	1.15±0.05

IC_50_ values were derived from 11-point CRCs on *Ae*Kir1 in Tl^+^ flux experiments performed in triplicate on two separate days.

**Figure 5:**
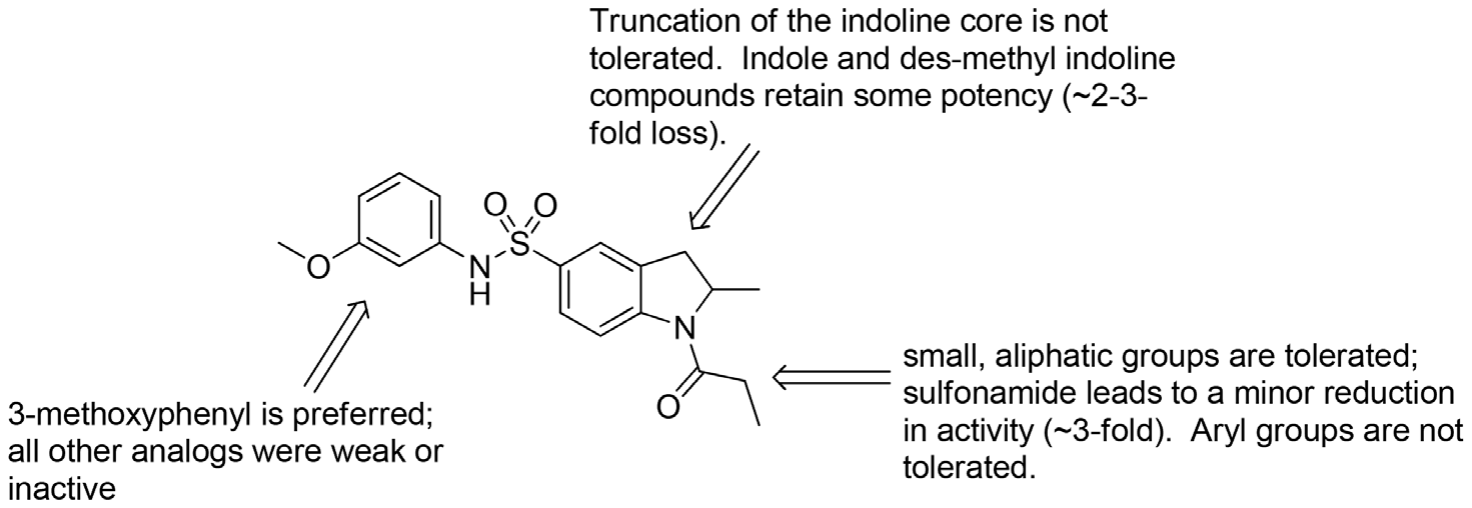
Summary of structure-activity relationship (SAR). Summary of observed SAR of over 100 analogs synthesized exploring all three regions of VU625.

Figure 5 summarizes the SAR observed for the VU625 scaffold. The left-hand sulfonamide portion offered the least amount of SAR traction as only the 3-methoxyphenyl (and weaker 2-methoxyphenyl) sulfonamide provided activity. Replacement with an amide, or substituting the 3-methoxyphenyl for other aryl groups all led to less potent compounds. Replacement of the proponamide in the southern fraction was tolerated as long as the substituent was small and aliphatic. Sulfonamides could be exchanged, although there was an observed ∼2-3-fold loss of activity. Lastly, the central core was also important for potency. Only very similar compounds such as indole and des-methyl indoline were tolerated.

### VU625-induced toxicity is increased by probenecid

**Figure 6:**
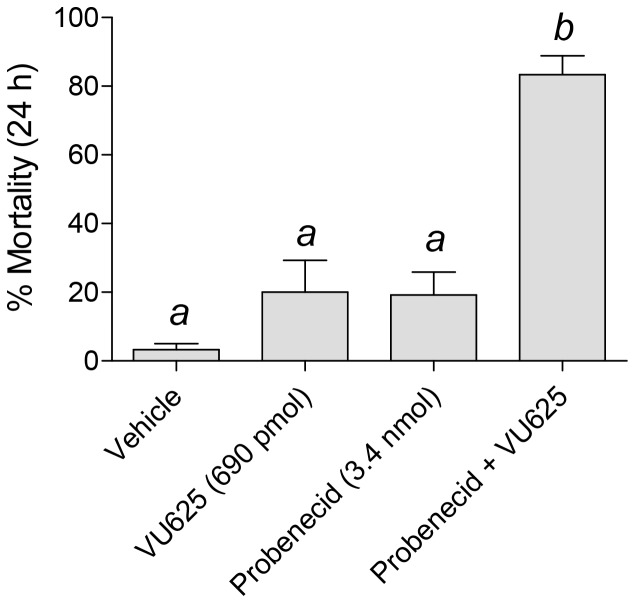
Effects of probenecid and VU625 on survival of adult female mosquitoes (*A. aegypti*). Percent mortality of mosquitoes at 24 h post-injection. Each mosquito was injected with 69 nl of the vehicle containing VU625 (10 mM), probenecid (50 mM), or both, to deliver the desired doses: 690 pmol of VU625, 3.4 nmol probenecid. *n* = 6–7 trials of 10 mosquitoes each per treatment. Lower-case letters indicate statistical categorization of the means as determined by a one-way ANOVA with a Newman-Keuls post-test (*P<0.05*).

Injection of the *Ae*Kir1 channel inhibitors VU573 or VU590 into the hemolymph of adult female *A. aegypti* mosquitoes leads to their incapacitation and/or death within 24 h [5], [6]. Surprisingly, injection of a high dose (i.e. 690 pmol per mosquito) of VU625, which is a more potent inhibitor of *Ae*Kir1 than VU573 and VU590, into the hemolymph of mosquitoes had no significant effects on mosquito behavior or survival within 24 h (Fig. 6). Thus, we hypothesized that the lack of in vivo effects could be due to poor bioavailability of VU625 as a result of metabolic detoxification and/or excretion by the mosquito.

We therefore tested whether probenecid, which is a broad-spectrum inhibitor of organic anion transporters (OATs) and ATP-binding cassette (ABC) transporters [24], [25], [26], would improve the efficacy of VU625. Interestingly, both probenecid and VU625 have a sulfonamide moiety in their chemical structure (Fig. S2). As shown in Fig. 6, the injection of probenecid (3.4 nmol per mosquito) along with VU625 (690 pmol per mosquito) significantly increases the toxicity of VU625 within 24 h compared to injection of VU625 or probenecid alone. The abdomens of these mosquitoes were not severely bloated and obvious sub-lethal effects (e.g., loss of flight) were not apparent.

**Figure 7:**
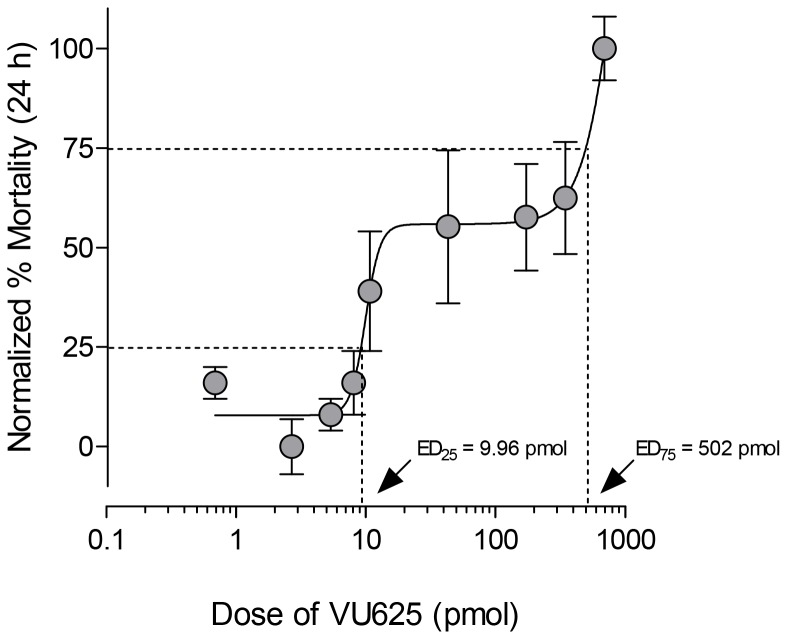
The dose-response curve of the toxic effects of VU625 on adult female mosquitoes (*A. aegypti*) is biphasic. Normalized percent mortality of mosquitoes at 24 h post-injection. Each mosquito was injected with 69 nL of the vehicle containing probenecid (50 mM) and an appropriate concentration of VU625 to deliver the doses of VU625 indicated and 3.4 nmol of probenecid. The ED_25_ and ED_75_ were determined by fitting a non-linear biphasic curve to the data. *n* = 3–4 trials of 10 mosquitoes each per dose.

We next sought to characterize the dose-response relationship of VU625 in mosquitoes when co-injected with a constant dose of probenecid (3.4 nmol per mosquito). As shown in Fig. 7, co-injection of VU625 with probenecid induces mortality in mosquitoes within 24 h in a biphasic manner with 25% and 75% efficacious doses (ED_25_ and ED_75_) of 9.96 pmol and 502 pmol, respectively. This biphasic dose-response relationship suggests the inhibition of at least two distinct molecular targets for which VU625 has different affinities, which is consistent with the inhibition of both *Ae*Kir1 and *Ae*Kir2B channels by VU625 (Fig. 3C).

### VU625-induced reduction of urine excretion is enhanced by probenecid

**Figure 8:**
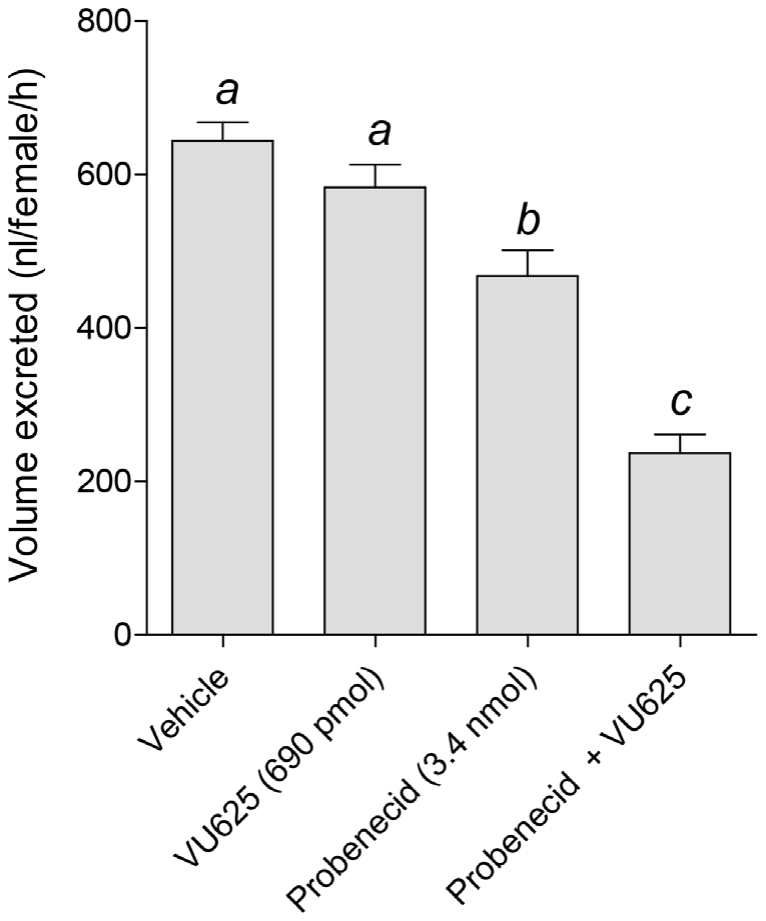
Effects of probenecid and VU625 on the in vivo excretory capacity of adult female mosquitoes (*A. aegypti*). Amount of urine excreted by mosquitoes 1 h after injection with 900 nL of the vehicle (K^+^-PBS_50_ containing 1.8% DMSO, 0.077% β-cyclodextrin, and 0.008% Solutol), or the vehicle containing VU625 (0.77 mM), probenecid (3.85 mM), or both, to deliver the desired doses: 690 pmol of VU625, 3.4 nmol probenecid. Values are means ±SEM; *n* = 6–18 trials of 5 mosquitoes per treatment. Lower-case letters indicate statistical categorization of the means as determined by a one-way ANOVA with a Newman-Keuls posttest (*P<0.05*).

We showed previously that pharmacological inhibition of *Ae*Kir1 with the small molecule inhibitors VU573 and VU590 leads to a decrease in the excretory capacity of *A. aegypti* mosquitoes after loading their hemolymph with 900 nl of a PBS vehicle [5] [6]. Therefore, we sought to similarly determine the effects of VU625 on mosquito excretory capacity. As shown in Fig. 8, mosquitoes injected with the PBS vehicle excreted 644±24.18 nL of urine within the next hour. Consistent with the toxicity studies, we found that adding VU625 (0.77 mM) to the vehicle, which delivers 690 pmol of VU625 per mosquito, did not significantly decrease the excretory capacity (583.3±29.52 nL/female) compared to the vehicle controls (Fig. 8). Interestingly, adding probenecid (50 mM) to the vehicle, which delivers 3.4 nmol of probenecid per mosquito, causes a small but significant reduction in excretory capacity to 467.8±33.53 nL/female, suggesting a potential role of probenecid-sensitive transporters in urine excretion. However, adding both VU625 (0.77 mM) and probenecid (50 mM) to the vehicle significantly decreases the excretory capacity the furthest to 236.7±24.53 nL/female.

## Discussion

Here, we report the discovery of VU625, the first sub-micromolar inhibitor of a mosquito Kir channel. VU625 is one of 283 confirmed *Ae*Kir1 inhibitors identified in a HTS of approximately 30,000 compounds from the VICB library. It was chosen for lead optimization based on its potency (IC_50_ = 96.8 nM), greater than 80-fold selectivity for the *Ae*Kir1 channel over 8 mammalian Kir channels, and clean ancillary pharmacology among a panel of 68 critical mammalian off-targets comprised of voltage-gated ion channels, ion transporters, and receptors (i.e., neurotransmitter, peptide, and G-protein coupled). VU625 is the most potent and selective mosquito Kir channel inhibitor reported to date.

This study provides proof-of-concept that conventional drug discovery approaches can be employed successfully to identify small-molecule tools for probing the physiology of insect Kir channels and potential lead compounds for insecticide development. A similar approach has been used recently in insecticide discovery efforts targeting mosquito G-protein coupled receptors [27].

VU625 exhibits inhibitory activity against both *Ae*Kir1 and *Ae*Kir2B, albeit with greater affinity for *Ae*Kir1. To date, we have reported the activity of two other small-molecule inhibitors of mosquito Kir channels that exhibit differential pharmacology. VU590 is a selective inhibitor of *Ae*Kir1 over *Ae*Kir2B, whereas VU573 inhibits *Ae*Kir1 and activates *Ae*Kir2B [6]. Thus, VU625 potentially represents a broad-spectrum, small-molecule blocker of mosquito Kir channels, pending the characterization of its effects on the other mosquito Kir channels (*Ae*Kir2A, *Ae*Kir2B' and *Ae*Kir3 channels), which to date have not yet been expressed functionally in a heterologous system ([12]; Denton and Piermarini, personal observations). Once the distinguishing pharmacological properties of each of these Kir channel inhibitors are fully characterized, they can potentially be employed to determine the relative contributions of Kir channel subtypes in the physiology of various mosquito tissues. This would provide an important chemical tool set to validate and complement studies of mosquito Kir channels that employ functional genetic approaches (e.g., RNA interference).

Given the superior in vitro potency of VU625 compared to the *Ae*Kir1 inhibitors VU573 [5] and VU590 [6], we expected VU625 to elicit superior in vivo toxicity. Thus, we were surprised when high doses of VU625 elicited no observable effects on mosquito survival or excretory capacity when injected directly into the hemolymph. Since mosquitoes have evolved robust protective mechanisms for detoxifying and excreting xenobiotics that would harm them otherwise [28], [29], we investigated whether the molecule may be detoxified and/or excreted.

Preliminary experiments with PBO did not improve the efficacy of VU625, suggesting that detoxification of the compound by cytochrome P450s is unlikely to contribute to its poor in vivo efficacy. The co-injection of VU625 with probenecid rescued not only its toxicity, but also its effects on excretory capacity, which suggests that VU625 is likely a substrate of OATs and/or ABC transporters in the mosquitoes and may be rendered ineffective in vivo through excretion. The potent toxicity of VU625 when co-injected with probenecid may be due to the ability of VU625 to inhibit at least two Kir channels, some of which are expressed in the central and peripheral nervous systems, such as Kir1 and Kir2B' [8], [12], [30], [31], and/or a synergistic effect of probenecid that maintains high circulating concentrations of VU625 by preventing its renal excretion. Indeed, it is conceivable that the sulfonamide moiety in the structures of VU625 and probenecid causes them to be substrates for OATs and/or ABC transporters. Overall, these findings highlight efficient xenobiotic transport mechanisms in mosquitoes that render a nanomolar inhibitor of *Ae*Kir1 (VU625) ineffective in vivo, even when introduced directly to the hemolymph. The tissues that contribute to the excretion of VU625 remain to be determined, but presumably involve the Malpighian tubules and/or gut [32], [33].

Lastly, the medicinal chemistry efforts put forth in the present study may be a valuable first step in determining which structural moieties are important for the excretion of VU625 by xenobiotic transporters and/or its in vivo activity in mosquitoes. Future studies should assess the in vivo efficacy and probenecid-mediated clearance of the VU625 analog series we generated to determine if any of these compounds exhibit potent toxicity in mosquitoes without probenecid.

## Perspectives

Here, we show a direct relationship between in vitro pharmacology and in vivo toxicity of VU625, which is consistent with our previous studies [5], [6] suggesting that Kir channel inhibitors are promising chemicals for insecticide development. To date, none of the Kir channel inhibitors we have reported (i.e. VU573, VU590, VU625) exhibit toxicity when applied to the cuticle (Piermarini, unpublished observations), which is a waxy, lipophilic structure that creates a physical barrier to insecticide permeation into the hemocoel of mosquitoes. This lack of topical activity severely limits the potential use of the present Kir channel inhibitors as active compounds for incorporation into insecticide-treated bed nets and indoor-residual sprays. The efficacy of common insecticides, such as permethrin, is dependent in part on their lipophilic nature [34], [35]. Thus, future chemistry efforts will focus on lipophilic inhibitors of Kir channels. Furthermore, prioritizing initial HTS ‘hits’ according to their hydrophobicity may facilitate the discovery of more suitable small-molecules compounds for insecticide development.

## Supporting Information

Figure S1 **Reagents and conditions: (A) TFAA, pyridine, 0°C; (B) ClSO_3_H, 40°C, 1 h; PCl_5_, rt; (C) 3-methoxyaniline, DIEA, rt; MeOH:10% NaOH (1∶1∶1); (D) pyridine, CH_2_Cl_2_, ClCOCH_2_CH_3._**
(TIF)

Figure S2 **VU625 and probenecid share a sulfonamide moiety.** The sulfonamide moiety contained in the chemical structure of VU625 and probenecid is shaded in blue.
(TIF)

Table S1 **Selectivity of VU625 against human Kir channels assessed in Tl^+^ flux assays.** ***n*** **= 2 independent experiments in triplicate.**
(DOCX)

Table S2 **Summary of results obtained from the activity of the VU625 compound in radioligand binding assays.** The significant results are highlighted in grey.
(DOCX)

Table S3 **SAR around the left-hand sulfonamide.** IC_50_ values were derived from 11-point CRCs on *Ae*Kir1 in Tl^+^ flux experiments performed in triplicate on two separate days.
(DOCX)
