# Correction: Discovery and Characterization of a Potent and Selective Inhibitor of Aedes aegypti Inward Rectifier Potassium Channels

**DOI:** 10.1371/journal.pone.0119883

**Published:** 2015-03-20

**Authors:** The PLOS ONE Staff

The ninth author’s name is spelled incorrectly. The correct name is: Sean R. Bollinger.
