# Supplementary figures and images for: Discovery and Characterization of a Potent and Selective Inhibitor of Aedes aegypti Inward Rectifier Potassium Channels
Source: PLoS One. 2014 Nov 6;9(11):e110772. doi: 10.1371/journal.pone.0110772 (PMC4222822; doi:10.1371/journal.pone.0110772)

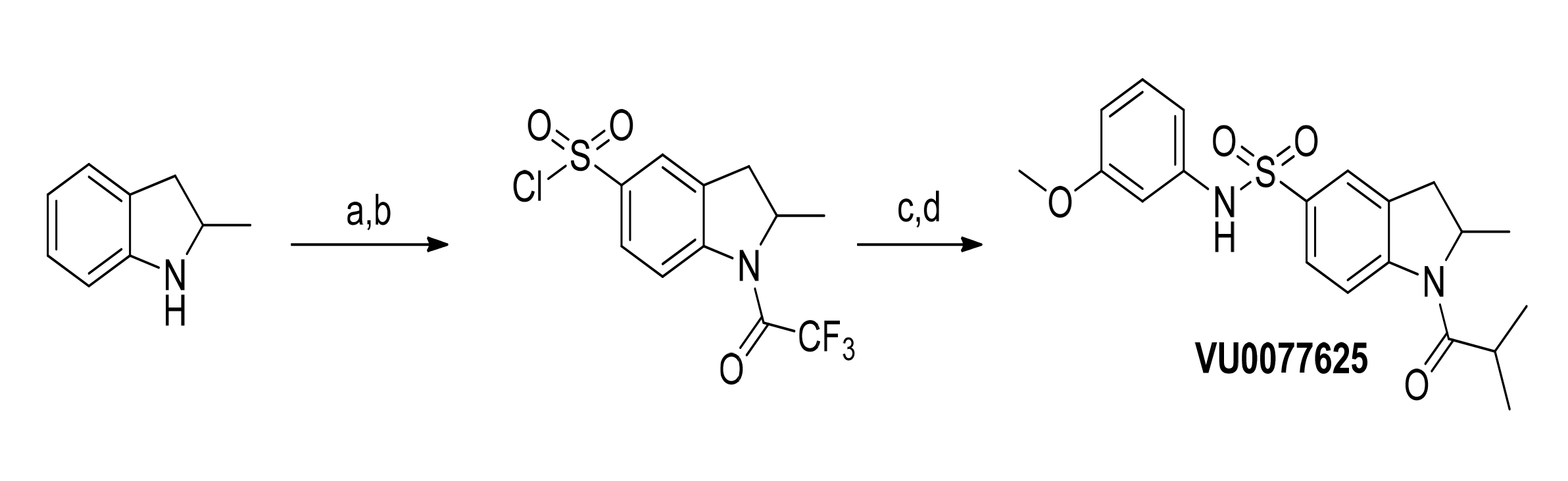

Supplement: Figure S1 — Reagents and conditions: (A) TFAA, pyridine, 0°C; (B) ClSO3H, 40°C, 1 h; PCl5, rt; (C) 3-methoxyaniline, DIEA, rt; MeOH:10% NaOH (1∶1∶1); (D) pyridine, CH2Cl2, ClCOCH2CH3. (TIF) [file pone.0110772.s001.tif]

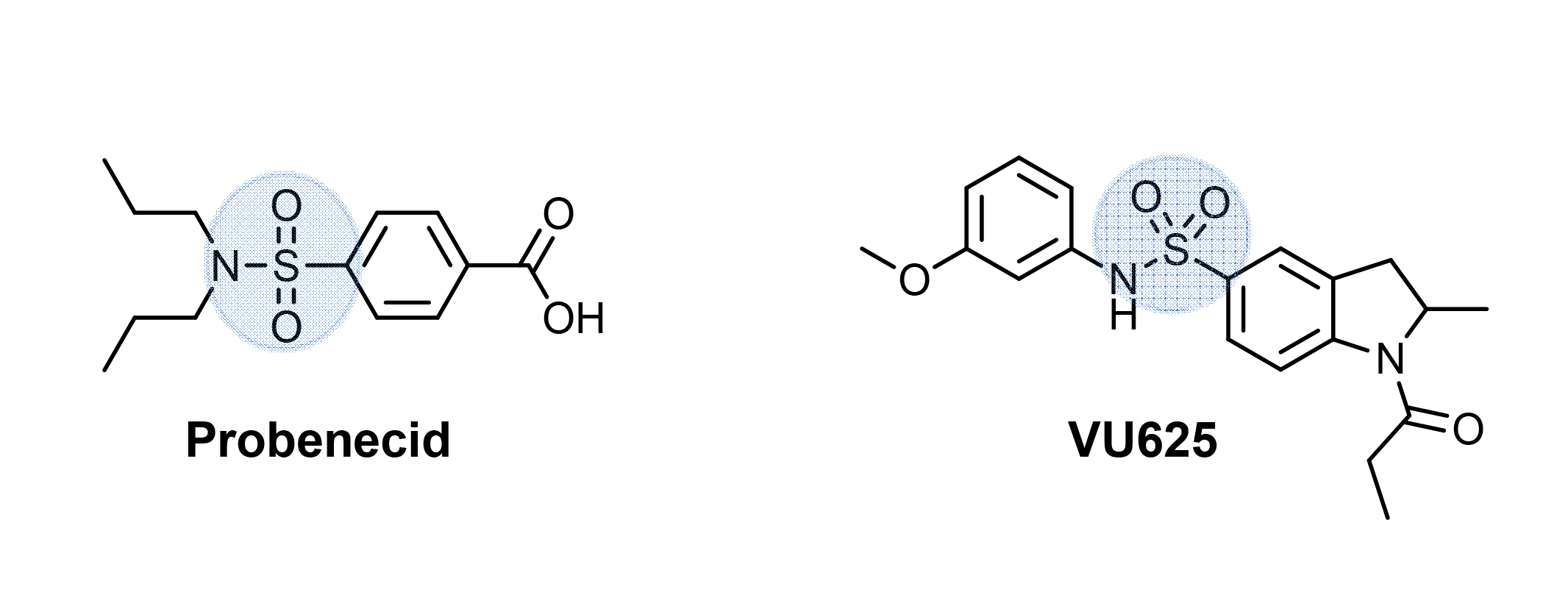

Supplement: Figure S2 — VU625 and probenecid share a sulfonamide moiety. The sulfonamide moiety contained in the chemical structure of VU625 and probenecid is shaded in blue. (TIF) [file pone.0110772.s002.tif]
