# Supplementary material for: Discovery and Characterization of a Potent and Selective Inhibitor of Aedes aegypti Inward Rectifier Potassium Channels
Source: PLoS One. 2014 Nov 6;9(11):e110772. doi: 10.1371/journal.pone.0110772 (PMC4222822; doi:10.1371/journal.pone.0110772)
Supplement: Table S1 — Selectivity of VU625 against human Kir channels assessed in Tl+ flux assays. n = 2 independent experiments in triplicate. (DOCX) [file pone.0110772.s003.docx]

**Supporting Information**

**Table S1.** Selectivity of VU625 against human Kir channels assessed in Tl^+^ flux assays. *n*= 2 independent experiments in triplicate

| **Kir channel** | **Inhibition of Tl^+^ flux (IC_50_, μM)** |
| --- | --- |
| Kir1.1 | >30 |
| Kir2.1 | >30 |
| Kir2.2 | >30 |
| Kir2.3 | >30 |
| Kir3.1/3.2 | 8.6 |
| Kir4.1 | >30 |
| Kir6.2/SUR1 | >30 |
| Kir7.1(M125R) | >30 |
