# Supplementary material for: Discovery and Characterization of a Potent and Selective Inhibitor of Aedes aegypti Inward Rectifier Potassium Channels
Source: PLoS One. 2014 Nov 6;9(11):e110772. doi: 10.1371/journal.pone.0110772 (PMC4222822; doi:10.1371/journal.pone.0110772)
Supplement: Table S2 — Summary of results obtained from the activity of the VU625 compound in radioligand binding assays. The significant results are highlighted in grey. (DOCX) [file pone.0110772.s004.docx]

**Supporting Information**

**Table S2.** Summary of results obtained from the activity of the VU625 compound in radioligand binding assays. The significant results are highlighted in grey.
