# Supplementary material for: Discovery and Characterization of a Potent and Selective Inhibitor of Aedes aegypti Inward Rectifier Potassium Channels
Source: PLoS One. 2014 Nov 6;9(11):e110772. doi: 10.1371/journal.pone.0110772 (PMC4222822; doi:10.1371/journal.pone.0110772)
Supplement: Table S3 — SAR around the left-hand sulfonamide. IC50 values were derived from 11-point CRCs on AeKir1 in Tl+ flux experiments performed in triplicate on two separate days. (DOCX) [file pone.0110772.s005.docx]

**Supporting Information**

**Table S3.** SAR around the left-hand sulfonamide. IC_50_ values were derived from 11-point CRCs on *Ae*Kir1 in Tl^+^ flux experiments performed in triplicate on two separate days.

| **Cmpd** | **VU** | **R** | **R^1^** | **R^2^** | **IC_50_ (μM)** |
| --- | --- | --- | --- | --- | --- |
| **5a** | VU0111100 | Me | H |  | 3.27 |
| **5b** | VU0111077 | Me | H |  | 3.44 |
| **3a** | VU0111158 | Me | H |  | 0.856 |
| **5c** | VU0077625 | Me | H |  | 0.338 |
| **5d** | VU0111108 | Me | H |  | 1.42 |
| **5e** | VU0472705 | Me | H |  | 1.15 |
| **5f** | VU0111168 | Me | Me |  | 2.16 |
| **5g** | VU0472699 | Me | H |  | 2.36 |
| **5h** | VU0472694 | Me | H |  | 3.49 |
| **5i** | VU0472696 | Me | H |  | 3.25 |
| **5j** | VU0472695 | Me | H |  | 1.36 |
| **5k** | VU0084675 | Me | H |  | 1.12 |
| **5l** | VU0111098 | Me | H |  | 4.94 |
| **5m** | VU0472698 | Me | H |  | 5.24 |
| **5n** | VU0472697 | Me | H |  | 3.54 |
| **5o** | VU0472702 | Me | H |  | 5.25 |
| **5p** | VU0472700 | Me | H |  | 1.55 |
| **5q** | VU0472703 | Me | H |  | 1.87 |
| **5r** | VU0472701 | Me | H |  | 1.97 |
| **5s** | VU0472704 | Me | H |  | 1.62 |
| **5t** | VU0110817 | H | H |  | >30 |
| **5u** | VU0110920 | H | H |  | >30 |
| **5v** | VU0485659 | H | H |  | >30 |
| **5w** | VU0144119 | H | H |  | >30 |
| **5x** | VU0485660 | H | H |  | 7.50 ± 0.70 |
| **5y** | VU0485652 | H | H |  | >30 |
| **5z** | VU0485653 | H | H |  | >30 |
| **5aa** | VU0485654 | H | H |  | >30 |
| **5bb** | VU0485655 | H | H |  | >30 |
| **5cc** | VU0077420 | H | H |  | >30 |
| **5dd** | VU0166512 | H | H |  | >30 |
| **5ee** | VU0486113 | H | H |  | >30 |
| **5ff** | VU0486114 | H | H |  | 3.47 ± 0.67 |
| **5gg** | VU0486115 | H | H |  | 1.40 ± 0.21 |
| **5hh** | VU0486121 | H | H |  | >30 |
| **5ii** | VU0486122 | H | H |  | >30 |
| **5jj** | VU0486123 | H | H |  | >30 |
| **5kk** | VU0486124 | H | H |  | >30 |
| **5ll** | VU0486125 | H | H |  | >30 |
| **5mm** | VU0486126 | H | H |  | 1.93 ± 0.07 |
| **5nn** | VU0486136 | H | H |  | >30 |
| **5oo** | VU0486137 | H | H |  | >30 |
| **5pp** | VU0486116 | H | Me |  | >30 |
| **5qq** | VU0486127 | H | Me |  | >30 |
